# Measuring the Effectiveness of COVID-19 Vaccines Used during a Surge of the Delta Variant of SARS-CoV-2 in Bangladesh: A Test-Negative Design Evaluation

**DOI:** 10.3390/vaccines10122069

**Published:** 2022-12-02

**Authors:** Farhana Khanam, Md Taufiqul Islam, Faisal Ahmmed, Shams Uddin Ahmed, Md Ismail Hossen, MdNazmul Hasan Rajib, Shahinur Haque, Prasanta Kumar Biswas, Imam Tauheed, K Zaman, Ahmed Nawsher Alam, Mallick Masum Billah, Monalisa, Shah Ali Akbar Ashrafi, Mohammed Ziaur Rahman, Omar Hamza Bin Manjur, Mokibul Hassan Afrad, S M Shamsuzzaman, Ahmed Abu Saleh, Mostafa Aziz Sumon, Asif Rashed, Md Taufiqur Rahman Bhuiyan, Fahima Chowdhury, Ashraful Islam Khan, Meerjady Sabrina Flora, Tahmina Shirin, John D. Clemens, Firdausi Qadri

**Affiliations:** 1 International Centre for Diarrhoeal Disease Research, Dhaka 1212, Bangladesh; 2 Institute for Epidemiology, Disease Control and Research, Dhaka 1212, Bangladesh; 3 Health Information Unit, Directorate General of Health Services, Health Services Division, Ministry of Health and Family Welfare, Dhaka 1212, Bangladesh; 4 Institute for Developing Science and Health Initiatives, Dhaka 1216, Bangladesh; 5 Dhaka Medical College and Hospital, Dhaka 1000, Bangladesh; 6 Bangabandhu Sheikh Mujib Medical University Hospital, Dhaka 1000, Bangladesh; 7 Kurmitola General Hospital, Dhaka 1206, Bangladesh; 8 Mugda Medical College and Hospital, Dhaka 1214, Bangladesh; 9 Directorate General of Health Services (DGHS), Mohakhali, Dhaka 1212, Bangladesh; 10 International Vaccine Institute, Seoul 08826, Republic of Korea; 11 UCLA Fielding School of Public Health, University of California, Los Angeles, Los Angeles, CA 90095-1772, USA

**Keywords:** Bangladesh, COVID-19 vaccines, Delta variant, vaccine effectiveness, test-negative design (TND)

## Abstract

Background: From May to December 2021, Bangladesh experienced a major surge in the Delta variant of SARS-CoV-2. The earlier rollout of several vaccines offered the opportunity to evaluate vaccine effectiveness against this variant. Methods: A prospective, test-negative case-control study was conducted in five large hospitals in Dhaka between September and December 2021. The subjects were patients of at least 18 years of age who presented themselves for care, suffering COVID-like symptoms of less than 10 days’ duration. The cases had PCR-confirmed infections with SARS-CoV-2, and up to 4 PCR test-negative controls were matched to each case, according to hospital, date of presentation, and age. Vaccine protection was assessed as being the association between the receipt of a complete course of vaccine and the occurrence of SARS-CoV-2 disease, with symptoms beginning at least 14 days after the final vaccine dose. Results: In total, 313 cases were matched to 1196 controls. The genotyping of case isolates revealed 99.6% to be the Delta variant. Receipt of any vaccine was associated with 12% (95% CI: −21 to 37, *p* = 0.423) protection against all episodes of SARS-CoV-2. Among the three vaccines for which protection was evaluable (Moderna (mRNA-1273); Sinopharm (Vero Cell-Inactivated); Serum Institute of India (ChAdOx1 nCoV-19)), only the Moderna vaccine was associated with significant protection (64%; 95% CI: 10 to 86, *p* = 0.029). Protection by the receipt of any vaccine against severe disease was 85% (95% CI: 27 to 97, *p* = 0.019), with protection estimates of 75% to 100% for the three vaccines. Conclusions: Vaccine protection against COVID-19 disease of any severity caused by the Delta variant was modest in magnitude and significant for only one of the three evaluable vaccines. In contrast, protection against severe disease was high in magnitude and consistent for all three vaccines. Because our findings are not in complete accord with evaluations of the same vaccines in more affluent settings, our study underscores the need for country-level COVID-19 vaccine evaluations in developing countries.

## 1. Introduction

The ongoing COVID-19 (coronavirus disease 2019) pandemic caused by severe acute respiratory syndrome coronavirus 2 (SARS-CoV-2) has been responsible for more than 416 million cases and around 6 million deaths to date [1]. The first approval for use of a COVID-19 vaccine following large-scale trials was given in the UK by the Medicines and Healthcare Products Regulatory Agency (MHRA) within 326 days after sequencing of the virus [2]. To date, mRNA vaccines have demonstrated high efficacy against symptomatic COVID-19 disease caused by most variants of concern [3,4], as have the adjuvanted recombinant spike protein vaccines. Adenovirus-vectored and killed whole-virion vaccines have conferred moderate protection [3,5]. The rollout of these vaccines has been highly segmented, with affluent, industrialized countries predominantly using doses of higher-efficacy vaccines, while developing countries, which have received later shipments and lower numbers of doses, have mostly deployed vaccines that confer lower efficacy. The Government of Bangladesh (GoB) rolled out its vaccination drive with the ChAdOx1 nCoV-19 vaccine, manufactured by the Serum Institute of India (Serum Institute of India (ChAdOx1 nCoV-19)) for frontline health workers on 27 January 2021, and for the general public on 7 February 2021. Subsequently, the GoB rolled out, in a staggered fashion, the Sinopharm (Vero Cell-Inactivated) vaccine, the Pfizer-BioNTech (BNT162b2) vaccine, the Moderna (mRNA-1273) vaccine, and the Sinovac (Vero Cell-Inactivated) vaccine, beginning on 16 June 2021 [6].

Effectiveness studies, performed after deployment, have been critical to monitoring the protection offered by vaccines as the pandemic has progressed, especially because vaccines have been challenged by many of the new variants that have arisen [7]. However, such studies have been considerably more frequent in affluent rather than in poor countries, leaving the latter with gaps in critical information about the real-world impact of the different constellations of vaccines used in these settings and among populations that have distinctive features that may impact vaccine protection in poorer settings [8]. Here, we report on an evaluation of the effectiveness of COVID-19 vaccines in Bangladesh, using the test-negative design, a variant of the case-control design, which has been successfully used in multiple vaccine evaluations in other countries [9,10,11].

## 2. Materials and Methods

### 2.1. Study Design, Site, and Vaccine Rollout

The study was carried out in four designated COVID-19 hospitals (Dhaka Medical College and Hospital, Kurmitola General Hospital, Mugda Medical College and Hospital, and Bangabandhu Sheikh Mujib Medical University Hospital) in Dhaka, Bangladesh, between 8 September 2021 and 29 December 2021. For this study, we prospectively enrolled patients who presented with COVID-19-like symptoms for RT-PCR testing, to identify the test-positive cases and test-negative controls.

### 2.2. Enrollment and Examination of Patients

Patients attending the outpatient isolation ward or inpatient unit of the designated hospitals with symptoms of SARS-CoV-2-like illness (fever, dry cough, tiredness, muscle or body aches, sore throat, diarrhea, conjunctivitis, headache, loss of taste or smell, rash on skin, or discoloration of the fingers and toes, difficulty breathing or shortness of breath, chest pain or pressure, or loss of speech or movement) and having performed an RT-PCR test for COVID-19 were approached for enrollment in the study. Patients aged ≥ 18 years, the age group targeted for vaccination before and during the study period, were considered to be eligible for the study if they had not been previously diagnosed with COVID-19 using RT-PCR testing and if the duration of symptoms before presentation was ≤10 days. Enrollment of patients was carried out in all health facilities from 8:30 to 14:00, six days a week, and informed written consent was obtained before enrollment in the study.

Upon presentation for testing, patients were approached and checked for eligibility criteria by a study nurse, and informed written consent was obtained. Each patient’s clinical information, including history and physical examination, was entered into structured data forms by a study physician. Disease at the time of presentation was classified as severe or not severe, using the WHO criteria [12]. Special attention was given to obtaining systematic data on demographic, clinical, and socioeconomic factors that were previously described as being related to the risk of COVID-19 [13,14,15]. Comorbidity was ascertained by history for individual conditions and was further characterized by a modified Charlson index, as described elsewhere [16]. Both the ascertainment of eligibility and the collection of presenting clinical data were obtained in a manner blinded to the subject’s status as a test-positive case or a test-negative control, as the results from PCR testing did not become available until the next day.

### 2.3. Ascertainment of Vaccination Status

At the time of presentation, the earlier receipt of the vaccine was ascertained by inspection of the official vaccination cards provided by the Government of Bangladesh (GoB) during vaccination. For persons not having cards, but giving a verbal history of vaccination, vaccination was confirmed by linking the national identification number of the subject to the centralized database of vaccination records of the management information system (MIS) of the GoB. Information was obtained about the date and brand of each dose. All ascertainment and linkage of vaccine histories and records was performed in a manner blinded to the patient’s RT-PCR results, and, thus, to the patient’s status as a case or a control. Complete vaccination was defined as the receipt of two doses of the same vaccine; subjects who had received heterologous two-dose regimens or who received the vaccine outside of Bangladesh were excluded.

### 2.4. Specimen Collection and Transportation to the Laboratories

At the time of presentation, a nasopharyngeal swab (NPS) specimen was collected from both nares using a swab, and the swab was then placed in a viral transport medium (VTM). The VTM, along with the swab stick, was placed in a cooler box with an ice pack (maintaining 2 to 8 °C) and transported to the Virology Laboratory of the International Centre for Diarrhoeal Disease Research, Bangladesh (icddr,b) for specimens from Dhaka Medical College and Hospital, Kurmitola General Hospital, and Mugda Medical College and Hospital, and sent to the Institute of Epidemiology and Disease Control Research (IEDCR) laboratory in the case of specimens collected from Bangabandhu Sheikh Mujib Medical University Hospital.

### 2.5. Laboratory Testing

Viral RNA from NPS was extracted using the Qiagen Viral RNA mini-kit (Qiagen, Germany), following the manufacturer’s instructions. Real-time PCR (RT-PCR) was performed by using CDC_N2, RdRp, and ORF3a primers and probes with the iTaq Universal Probes Reaction Master Mix (Biorad, CA, USA), iScript Reverse Transcriptase. The RNaseP housekeeping-gene RNA level was performed to determine the quality of the NPS sample. A positive RT-PCR result with a ≤40-cycle threshold (CT) value was taken as positive. Other results were classified as negative. Once the RT-PCR test result was available, the laboratory result was entered digitally into a special form. RT-PCR-positive samples with a ≤30 CT value were also subjected to whole-genome sequencing using the MinION sequencing platform, carried out at the Institute for Developing Science and Health Initiatives (ideSHi) laboratory. Lineages were assigned by the SARS-CoV-2 pangolin (github.com/cov-lineages/pangolin; accessed on 15 January 2022) classification system.

### 2.6. Selection of Cases and Controls

On receiving the RT-PCR results, eligible cases (RT-PCR-positive COVID-19 patients) and controls (RT-PCR-negative patients) were listed for each day, and controls were selected for each case at each hospital at a ratio of up to 4:1 by a statistician blinded to the vaccination status of the subjects. Controls were matched to cases by site and date of presentation for care and age on the date of testing (18–30 years, 31–60 years, and >60 years) and were randomly sampled if the number of eligible, matched control patients exceeded 4 for a given case. If 4 matched controls could not be found for a case on the date of presentation, the sampling frame was progressively widened, beginning with the day before, then the day after, and so on until a frame of ±3 days was reached. The selection of cases followed the principles of incidence density sampling: cases were selected in sequential order and could not be later resampled as cases, but controls could later be resampled as cases or controls.

### 2.7. Follow-Up of Patients

For the purposes of this analysis, we incorporated data on mortality from a follow-up conducted 30 days after enrollment.

### 2.8. Sample Size Estimation and Statistical Analysis

Our primary goal, formally documented in a statistical analysis plan that was finalized before analyzing the data, was to assess the overall impact of all vaccines rolled out in Bangladesh upon all episodes of detected COVID disease. Accordingly, our sample-size calculation was designed to detect 60% protection against symptomatic SARS-CoV-2 disease by the receipt of a complete regimen of any COVID-19 vaccine at *p* < 0.05 (two-tailed) with at least 80% power, assuming that 12% of controls were vaccinated, with 4 controls per case. These assumptions yielded a required size of 323 cases and 1292 controls. Our primary analysis examined the protection given by a complete two-dose series of any homologous regimen of vaccines administered by the GoB at least 14 days before presentation for symptomatic episodes of RT-PCR-confirmed SARS-CoV-2 disease of any level of severity. In secondary analyses, also pre-specified in the statistical analysis plan, we evaluated the protection offered by complete vaccine regimens against severe SARS-CoV-2 disease, as well as the protection offered by a complete regimen by duration since the receipt of the second dose. Severe disease in these analyses was defined as “presenting severe disease” at the time of presentation for testing, using the WHO criteria [12], or “ultimate severe disease”, including patients either exhibiting severe disease or dying within the 30 days following presentation. We were not able to power our study to evaluate the protection offered by the individual types of vaccines used in Bangladesh. However, because different vaccines have conferred different levels of protection in other studies, we also conducted exploratory analyses of protection by each vaccine.

Bivariate analyses were performed in a matched fashion and conducted between relevant demographic, behavioral, and clinical variables and disease status using bivariate conditional logistic regression models. For the evaluation of vaccine protection, we used conditional logistic regression, considering the matched case-control status as the outcome and vaccination status and selected covariates as independent variables, taking the non-receipt of vaccine as the referent category for the assessment of vaccine protection. For unmatched analyses, we compared cases and controls with the chi-square test for the categorical variables and Student’s *t*-test for the dimensional variables and used unconditional logistic regression models to estimate vaccine protection. The odds ratios for the associations between vaccination and disease status were estimated by exponentiation of the coefficient for vaccination from the fitted models, and the standard error of the coefficient was used to calculate 95% confidence intervals for the estimated effect. Protective effectiveness was calculated as ((1 − odds ratio) × 100%)). For evaluation of vaccine protection against all disease episodes, severity at presentation was forced into the models as an independent variable, in order to help protect against healthcare utilization bias [15]. Other potentially confounding variables were introduced as independent variables in the models if the bivariate associations had *p*-values ≤ 0.10 (two-tailed), and if the associations remained significant at *p* < 0.05 (two-tailed) in the stepwise variable selection models. This threshold was also applied when judging the significance of protective associations between vaccination and disease status.

Because of the paucity of severely ill SARS-CoV-2 patients enrolled in the study, we also conducted an exploratory analysis comparing verified cases presenting severe disease versus controls presenting severe disease, disregarding the matched selection. The restriction of cases and controls to those presenting severe disease was employed to help further safeguard against residual healthcare utilization bias, as both the uptake of vaccination and the use of healthcare facilities may differ in those presenting with severe versus non-severe disease. In this analysis, unconditional logistic regression models were fitted, and the matching variables for the site of presentation, time of presentation (the month was used), and age at presentation were forced into the models as covariates. All analyses were performed after a formal data lock and according to the analyses outlined in a statistical analysis plan. We used R statistical software (version 4.10) for data analyses. The clogit package of R was used to estimate the conditional logistic regression coefficients by maximizing the conditional likelihood.

## 3. Ethical Review

Before initiation of the study, ethical approval from the Research and Ethical Review Committees of the icddr,b was obtained. Informed written consent was obtained from the participants prior to enrollment in the study.

## 4. Results

### 4.1. Assembly of the Study Population

**Figure 1:**
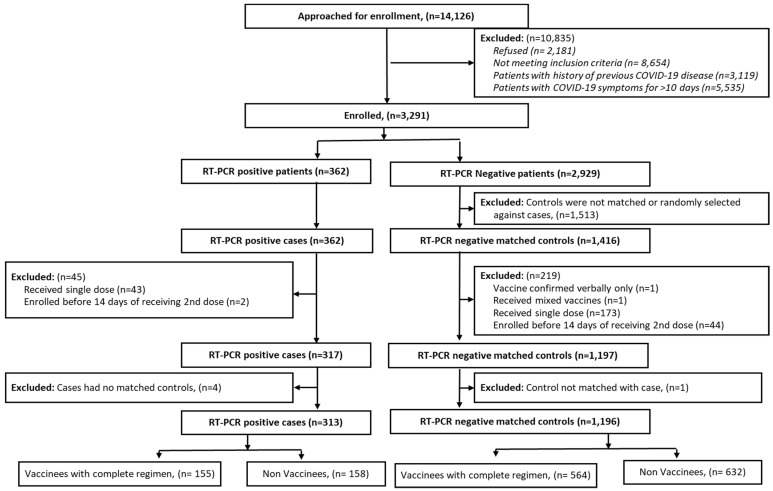
CONSORT diagram for the assembly of the study population.

We approached 14,126 patients attending the hospitals in question for COVID-19 testing. Of these, 3291 eligible patients were enrolled after giving their informed consent. Among these subjects, we identified 362 RT-PCR-positive cases and were able to select 1416 matched RT-PCR-negative controls. Finally, 313 cases and 1196 matched controls were included in the analysis, after restricting the inclusion of vaccinated subjects to those who had received an adequately documented, complete, homologous vaccine regimen in Bangladesh at least 14 days before presentation for testing (Figure 1). No subjects had received a post-primary booster dose of the vaccine. Whole-genome sequencing revealed 274 (99.6%) sequenced RT-PCR positive cases to be the Delta variant (B.1.617.2) and one case (0.4%) to be the Omicron variant (B.1.1.529). 

### 4.2. Comparability of Cases and Controls

In simple comparisons with matched cases and controls (Table 1), we found that being of the female sex, of non-Muslim religion, and having membership in smaller and wealthier households, a higher body mass index (BMI), and a history of non-smoking were associated with SARS-CoV-2 disease. Further analysis found the relationship between sex, smoking, and the occurrence of SARS-CoV-2 disease to be highly confounded since no females were smokers. Among several comorbid conditions ascertained by history, only diabetes mellitus was associated with COVID-19 disease. At presentation, the positive cases were more severely ill than controls; positive cases were more likely than controls to be classified as ultimately severe at 30 days of post-presentation follow-up, although similar proportions (3 (1%) of the 313 cases and 10 (1%) of the 1196 controls) had died by the 30-day follow-up. Comparisons of ultimately severe cases and their matched controls (ultimately severe or not) only showed significant associations with being female and a non-smoker. Table S1 in the Supplementary Material provides simple comparisons of cases and controls that were classified as severe at presentation, disregarding the match. Baseline features distinguishing these cases from the controls included female sex, a negative smoking history, and a higher BMI.

### 4.3. Protection by Receipt of Complete Regimens of Any Vaccine Deployed against COVID-19

The adjusted protective effectiveness (PE) value against all episodes of COVID-19 disease conferred by the receipt of a complete regimen of any vaccine rolled out was 12% (95% CI: −21 to 37, *p* = 0.423) (Table 2). For COVID-19 episodes classified as ultimately severe, the protection was substantially greater (85%; 95% CI: 27 to 97, *p* = 0.019) (Table 3). Results for protection against severe disease by the aggregate of the vaccines rolled out were similar when we limited the evaluation to cases and controls presenting with severe disease (80%; 95% CI; −3 to 98, *p* = 0.079) (Supplementary Table S2).

**Figure 2:**
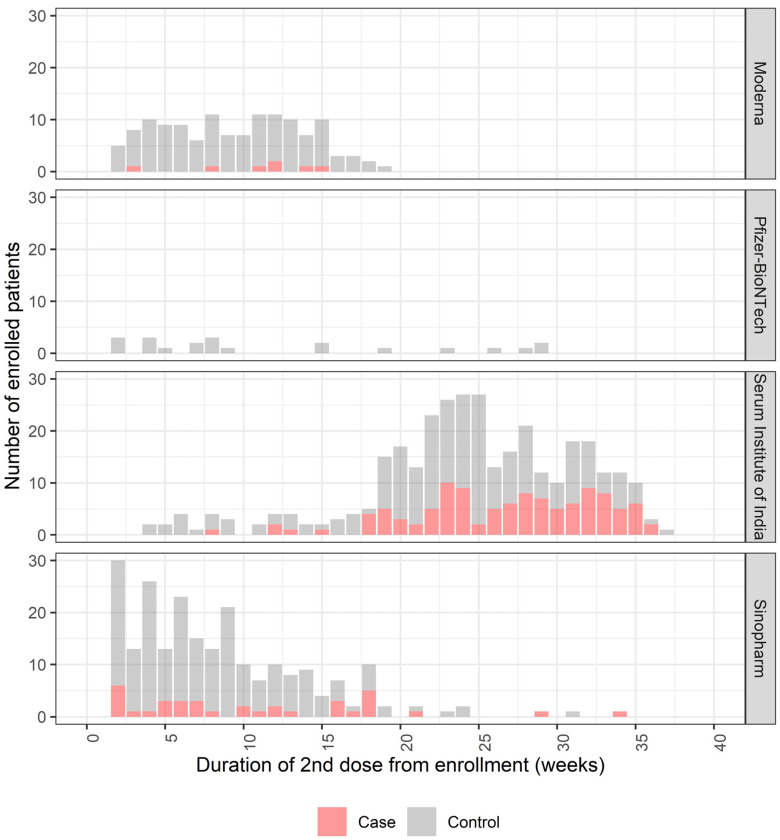
Vaccination of cases and controls by time since the second dose of vaccine in those with complete regimens (date of presentation at least 14 days after the second dose).

To evaluate vaccine protection by time since the receipt of the second dose, we approximately bisected the period of enrollment into the period of ≤19 weeks versus the period of >19 weeks after the second dose and retained the matched selection strategy. Most positive cases and controls were vaccinated with the Serum Institute of India (ChAdOx1 nCoV-19) vaccine and were enrolled more than 19 weeks before presentation; most cases and controls were vaccinated with either the Moderna (mRNA-1273) or Sinopharm (Vero Cell-inactivated) vaccine and were enrolled within 19 weeks before presentation (Figure 2). We found 42% (95% CI: 11 to 62, *p* = 0.013) of protection by any vaccine against symptomatic COVID-19 disease in those participants who were enrolled within 19 weeks of the second dose (Table 4). Beyond 19 weeks after the second dose, no protection was observed when the receipt of any vaccine was considered (−35%; 95% CI: −111 to 13, *p* = 0.182).

### 4.4. Protection by Receipt of Complete Regimens of the Individual Types of Vaccine Deployed against COVID-19

In the exploratory analyses, we assessed the protection conferred by the receipt of a complete regimen of each type of vaccine that was rolled out, while recognizing that the study was originally powered only to evaluate the protection conferred by the aggregate of vaccines rolled out. Individually, only the Moderna (mRNA-1273) vaccine showed significant protection against all episodes of COVID-19 disease (64%; 95% CI: 10 to 86, *p* = 0.029). While there was a suggestion of protection by the Sinopharm (Vero Cell-Inactivated) vaccine (29%; 95% CI: −22 to 58, *p* = 0.213), the point estimate for the Serum Institute of India vaccine (ChAdOx1 nCoV-19) revealed no protection (−45%; 95% CI: −119 to 4, *p* = 0.078) (Table 2).

For ultimately severe COVID-19 disease, each vaccine was associated with the point estimates of protection from 75% to 100%, though confidence intervals were very wide due to sample-size limitations (Table 3). When we repeated the analyses for all cases and controls who had severe disease at presentation, we found high point estimates of protection for receipt of the Sinopharm (Vero Cell-Inactivated) (87%; 95% CI: −8 to 99, *p* = 0.098) and Moderna (mRNA-1273) vaccines (100%; 95% CI: −Inf to 100%, *p* = 0.996), but not for the Serum Institute of India vaccine (−41%; 95% CI: −4220 to 96, *p* = 0.827). However, wide 95% confidence intervals again limited our interpretation of these estimates (Table S2 in the Supplementary Materials).

Finally, when we evaluated vaccine protection against any symptomatic disease by duration since the second dose, the Moderna (mRNA-1273) vaccine exhibited 64% (95% CI: 10 to 86, *p* = 0.029) protection, the Sinopharm (Vero Cell-Inactivated) vaccine, 34% protection (95% CI: −16 to 62, *p* = 0.147) and the Serum Institute of India (ChAdOx1 nCoV-19) vaccine, 3% protection (95% CI: −129 to 59, *p* = 0.940) during the first 19 weeks. Beyond 19 weeks after the second dose, there was no subject who had been vaccinated with the Moderna (mRNA 1273) vaccine, and no protection was observed for the Serum Institute of India (ChAdOx1 nCoV-19) or the Sinopharm (Vero Cell-Inactivated) vaccines (Table 4).

## 5. Discussion

During a period in which the Delta variant accounted for virtually all SARS-CoV-2 infections, the receipt of a complete regimen of the aggregate of vaccines deployed in Bangladesh was associated with little evidence of protection against disease of any severity among patients seeking treatment. However, this overall result belied a much more complex pattern of vaccine protection; the initial 19 weeks after the receipt of any vaccine was associated with significant protection (42%; 95% CI: 11 to 62), but that protection disappeared in the subsequent 19 weeks (−35%; 95% CI: −111 to 13).

In contrast, protection by any deployed vaccine against ultimately severe COVID-19 disease over the entire study interval was of high grade (85%; 95% CI: 27 to 97), a result that was corroborated when cases and controls were limited to patients who presented with severe disease, a tactic designed to help remove the residual bias due to differential health-seeking behavior in cases versus controls.

Because protection by different COVID-19 vaccines has been shown to differ in past studies, we conducted exploratory analyses of these relationships for the different vaccines deployed. Over the entire study interval, only the Moderna (mRNA-1273) vaccine was associated with significant protection against all COVID-19 disease episodes, albeit of moderate magnitude (64%; 95% CI: 10 to 86). Conversely, the Serum Institute of India (ChAdOx1 nCov-19) vaccine failed to exhibit any protection. A wide confidence interval precluded inferences about protection by the Sinopharm (Vero-Cell Inactivated) vaccine. Although confidence intervals were wide, point estimates of protection against ultimate severe COVID-19 disease were high for all three vaccines (75% to 100%).

It is important to consider several potential limitations of our study. As a study based in just five hospitals serving an urban population of nearly 20 million people who have many other sources of care available, our findings cannot be taken as a basis to generalize for the entire city of Dhaka. However, the use of the sampling frame provided by the test-negative design allowed the selection of controls who could reasonably be considered to represent persons who would have been selected as cases had they developed and sought care for COVID-19. This feature, in conjunction with exhaustive analytical control for potential confounding variables, prospective conduct of the study, determination of eligibility, and acquisition of informed consent without knowledge of the PCR status of subjects, and the ascertainment of vaccination status based on objectively verified sources, obtained in a fashion without knowledge of subjects’ status as cases or controls, likely ensured a high level of internal study validity. Moreover, extensive genotyping of SARS-CoV-2 isolates from the cases in our study allowed us to conclude that our findings pertain to the Delta variant, which was responsible for disease in virtually all cases. Another limitation of our study was that it was powered for the assessment of receipt of any vaccine, rather than of individual vaccines, and was not well-powered to evaluate protection against clinically severe SARS-CoV-2 disease, accounting for a wide 95% confidence interval and high *p*-values. Nonetheless, our exploratory analyses did reveal statistically significant protection by the Moderna vaccine against disease of any severity, and high-grade, statistically significant protection by the receipt of any vaccine against severe disease, with consistently high point estimates of protection for each of the three vaccines in predominant use. Finally, although the Pfizer-BioNtech (BNT162b2 mRNA) vaccine and the Sinovac (Vero Cell-Inactivated) vaccine have been introduced into Bangladesh, their use was not common enough for exploratory analyses of protection by each vaccine.

Our estimates of vaccine protection against episodes of SARS-CoV-2 disease of any severity were generally lower than those reported elsewhere for the Delta variant. We found 64% protection from the Moderna (mRNA-1273) vaccine against symptomatic SARS-CoV-2 infection, whereas, in a study conducted in the United States, the vaccine showed 93% protection against symptomatic disease caused by the Delta variant [17]. A test-negative design study conducted in the UK reported 31% effectiveness against symptomatic infection due to the Delta variant after one dose of either the Pfizer (BNT162b2) or the Astra-Zeneca (ChAdOx1) vaccine, whereas, after two doses, the effectiveness was 88% and 67% for the two vaccines, respectively [18]. In contrast, our study found no evidence of protection by the Serum Institute of India (ChAdOx1) vaccine, which was identical to the AstraZeneca (ChAdOx1) vaccine.

In contrast, our estimates of vaccine protection against severe SARS-CoV-2 disease are largely consistent with estimates of vaccine protection against the Delta variant reported elsewhere. Our evaluation found a point estimate of 75% protection by the Sinopharm (Vero Cell-Inactivated) vaccine against ultimate severe disease; the vaccine conferred 95% protection against hospitalization and critical admissions caused by the Delta variant in the United Arab Emirates [19]. In India, the Serum Institute of India (ChAdOx1 nCoV-19) vaccine exhibited 95% protection against moderately severe disease during the period when the Delta variant was predominant, similar to the 86% observed in our study [20]. A test-negative design study of a complete course of the Moderna (mRNA-1273) vaccine revealed 96% protection against any severe, critical, or fatal cases of COVID-19 disease due to the Delta variant in Qatar, similar to the 100% point estimate of protection observed in our study [21].

Our study is the first to evaluate the protective effectiveness of the SARS-CoV-2 vaccines deployed in Bangladesh. The fact that our study, which used the same test-negative design approach widely employed for real-world evaluations of COVID-19 vaccines worldwide, revealed lower estimates of vaccine protection against COVID-19 diseases of any severity but showed similar protection against severe disease in comparison with studies of the same vaccines conducted in other countries, may in part reflect the wide confidence intervals of our estimates for the exploratory analyses, but may also illustrate that the levels of vaccine protection against the Delta variant may vary according to the geographical context of the evaluation. These findings underscore the importance of the continued country-level monitoring of vaccine effectiveness as the SARS-CoV-2 pandemic evolves and will inform future models of SARS-CoV-2 vaccine deployments. The Delta surge in Bangladesh has now passed, and we are continuing the study during the current surge by the Omicron variant, which likely will offer additional insights.

## Figures and Tables

**Table 1:** Comparability of cases and controls.

Name of Variables	Parameters	Cases (*n* = 313)	Matched Controls (*n* = 1196)	*p*-Value ^¶^	Ultimate Severe Cases * (*n* = 30)	Matched Controls (*n* = 104)	*p*-Value ^¶^
Month-wise enrolled participants	September	52 (16.6)	210 (17.6)	0.052	9 (30)	31 (29.8)	-
	October	95 (30.4)	397 (33.2)		13 (43.3)	46 (44.2)	
	November	88 (28.1)	316 (26.4)		7 (23.3)	24 (23.1)	
	December	78 (24.9)	273 (22.8)		1 (3.3)	3 (2.9)	
Age in years (mean ± SD)		41.3 ± 15.8	41.1 ± 15.5	0.900	54.9 ± 18.8	52.7 ± 15.5	0.100
Age groups	18–30 years	93 (29.7%)	369 (30.9%)	-	2 (6.7%)	6 (5.8%)	-
	31–60 years	176 (56.2%)	660 (55.2%)		15 (50%)	54 (51.9%)	
	61+ years	44 (14.1%)	167 (14.0%)		13 (43.3%)	44 (42.3%)	
Sex	Female	138 (44.1%)	406 (33.9%)	<0.001	18 (60%)	34 (32.7%)	0.008
	Male	175 (55.9%)	790 (66.1%)		12 (40%)	70 (67.3%)	
Religion	Non-Muslim	31 (9.9%)	60 (5.0%)	0.003	0 (0%)	3 (2.9%)	0.998
	Muslim	282 (90.1%)	1136 (95.0%)		30 (100%)	101 (97.1%)	
Body mass index (BMI)	KG/M^2^	24.8 ± 4.1	24 ± 4.1	0.005	24.5 ± 5	23.2 ± 3.8	0.096
HH ^†^ members	Count	4.3 ± 2	4.6 ± 2.1	0.056	4.7 ± 2	5.2 ± 2.5	0.341
HH ^†^ Income	BD Taka	52,530.4 ± 47,923.9	37,528 ± 51,304.2	<0.001	27,000 ± 16,175.8	31,781.7 ± 32,738.5	0.476
Smoker ^‡^	No	264 (84.3%)	902 (75.4%)	0.001	27 (90%)	71 (68.3%)	0.025
	Yes	49 (15.7%)	294 (24.6%)		3 (10%)	33 (31.7%)	
Ultimate severity *	No	283 (90.4%)	1141 (95.4%)	<0.001	0 (0.0%)	86 (82.7%)	0.998
	Yes	30 (9.6%)	55 (4.6%)		30 (100%)	18 (17.3%)	
Severity at presentation	No	286 (91.4)	1151 (96.2)	<0.001	3 (10%)	90 (86.5%)	<0.001
	Yes	27 (8.6)	45 (3.8)		27 (90%)	14 (13.5%)	
Heart disease ^§^	No	300 (95.8%)	1143 (95.6%)	0.822	28 (93.3%)	98 (94.2%)	0.865
	Yes	13 (4.2%)	53 (4.4%)		2 (6.7%)	6 (5.8%)	
Hypertension ^§^	No	252 (80.5%)	1007 (84.2%)	0.201	23 (76.7%)	81 (77.9%)	0.972
	Yes	61 (19.5%)	189 (15.8%)		7 (23.3%)	23 (22.1%)	
Lung disease ^§^	No	304 (97.1%)	1169 (97.7%)	0.524	30 (100%)	103 (99.0%)	0.998
	Yes	9 (2.9%)	27 (2.3%)		0 (0%)	1 (1.0%)	
Diabetes mellitus ^§^	No	270 (86.3%)	1080 (90.3%)	0.086	25 (83.3%)	97 (93.3%)	0.086
	Yes	43 (13.7%)	116 (9.7%)		5 (16.7%)	7 (6.7%)	
Stomach disease ^§^	No	311 (99.4%)	1182 (98.8%)	0.455	30 (100%)	102 (98.1%)	0.998
	Yes	2 (0.6%)	14 (1.2%)		0 (0%)	2 (1.9%)	
Kidney disease ^§^	No	312 (99.7%)	1186 (99.2%)	0.407	29 (96.7%)	102 (98.1%)	0.571
	Yes	1 (0.3%)	10 (0.8%)		1 (3.3%)	2 (1.9%)	
Liver disease ^§^	No	313 (100%)	1189 (99.4%)	0.995	0 (0%)	0 (0.0%)	-
	Yes	0 (0%)	7 (0.6%)		30 (100%)	104 (100%)	
Anaemia ^§^	No	313 (100%)	1194 (99.8%)	0.995	0 (0%)	0 (0.0%)	-
	Yes	0 (0.0%)	2 (0.2%)		30 (100%)	104 (100%)	
Cancer ^§^	No	312 (99.7%)	1194 (99.8%)	0.628	29 (96.7%)	103 (99.0%)	0.381
	Yes	1 (0.3%)	2 (0.2%)		1 (3.3%)	1 (1.0%)	
Depression ^§^	No	311 (99.4%)	1191 (99.6%)	0.666	29 (96.7%)	103 (99.0%)	0.327
	Yes	2 (0.6%)	5 (0.4%)		1 (3.3%)	1 (1.0%)	
Osteoarthritis ^§^	No	313 (100%)	1194 (99.8%)	0.996	30 (100%)	103 (99%)	0.998
	Yes	0 (0.0%)	2 (0.2%)		0 (0%)	1 (1.0%)	
Modified Charlson Comorbidity Index ^||^	Score	0.9 ± 1.7	0.8 ± 1.6	0.261	1.6 ± 2.1	1.1 ± 1.9	0.153

* Ultimate severity includes severity at presentation and death within 30 days of enrollment. ^†^ HH represents household. ^‡^ A person who smokes tobacco regularly. ^§^ Ascertained by history. ^||^ Modified Charlson Comorbidity Index [16]. ^¶^ *p*-value of the conditional logistic regression model (Wald test).

**Table 2:** Protection against any symptomatic COVID-19 disease by the receipt of complete regimens of vaccines *.

	Cases		Matched Controls		Protective Effectiveness (PE) % (95% Confidence Interval (CI))			
Vaccines	Vaccinees	Non-Vaccinees	Vaccinees	Non-Vaccinees	Crude PE	*p*-Value	Adjusted ^†^ PE	*p*-Value
Any vaccine ^‡^	155	158	564	632	−8 (−44, 19)	0.589	12 (−21, 37) ^ƭ^	0.423
Serum Institute of India (ChAdOx1 nCoV-19) ^§^	105	156	179	484	−79 (−156, −26)	0.001	−45 (−119, 4) ^ƭ^	0.078
Sinopharm (Vero Cell-Inactivated) ^||^	35	153	100	396	23 (−28, 54)	0.309	29 (−22, 58) ^Ƭ^	0.213
Moderna (mRNA-1273) ^¶^	6	150	42	357	69 (25, 87)	0.010	64 (10,86) ^ƾ^	0.029

* Received a second dose of vaccine at least 14 days before presentation. ^†^ Adjusted by forced variable (severity at presentation) with other covariates significantly associated at *p* < 0.05 in the stepwise model: ^ƭ^ study month, gender, religion, household size, and household income. ^Ƭ^ Body mass index and smoking status. ^ƾ^ Gender and smoking status. ^‡^ Any vaccine (Serum Institute of India (ChAdOx1 nCoV-19), Sinopharm (Vero Cell-Inactivated), Moderna (mRNA-1273), Pfizer-BioNTech (BNT162b2), and Sinovac (Vero Cell-Inactivated). ^§^ Participants with only Serum Institute of India-ChAdOx1 nCoV-19 vaccination or non-vaccinees (cases = 270; matched controls = 663); 9 cases were excluded since no matched controls were available. ^||^ Participants with only Sinopharm (Vero Cell-Inactivated) vaccination or non-vaccines (cases = 194; matched controls = 496); 6 cases excluded since no matched controls were available. ^¶^ Participants with only Moderna mRNA-1273 vaccination or non-vaccinees (cases = 165; matched controls = 399); 9 cases excluded since no matched controls were available.

**Table 3:** Protection against ultimate severe COVID-19 disease by the receipt of complete regimens of vaccines *.

	Ultimately Severe Cases		Matched Controls		Protective Effectiveness (PE) % (95% Confidence Interval, CI)			
Vaccines	Vaccinees	Non-Vaccinees	Vaccinees	Non-Vaccinees	Crude PE	*p*-Value	Adjusted ^†^ PE	*p*-Value
Any vaccine ^‡^	3	27	34	70	86 (32, 97)	0.015	85 (27, 97)	0.019
Serum Institute of India (ChAdOx1 nCoV-19) ^§^	2	27	12	66	85 (−39, 98)	0.095	86 (−23, 98)	0.076
Sinopharm (Vero Cell-Inactivated) ^||^	1	26	12	68	79 (−77, 98)	0.151	75 (−124, 97)	0.214
Moderna (mRNA-1273) ^¶^	0	26	8	64	100 (−Inf, 100)	0.998	100 (−Inf, 100)	0.999

* Received a second dose of vaccine at least 14 days before presentation. ^†^ Adjusted by covariates gender and smoking status are significantly associated at *p* < 0.05 in the bivariate conditional logistic regression model. ^‡^ Any vaccine (Serum Institute of India (ChAdOx1 nCoV-19), Sinopharm (Vero Cell-Inactivated), Moderna (mRNA-1273), and Sinovac (Vero Cell-Inactivated)). ^§^ Participants who received only the Serum Institute of India-ChAdOx1 nCoV-19 vaccine or non-vaccinees (severe cases = 29; matched controls = 78). ^||^ Participants who received only Sinopharm (Vero Cell-Inactivated) vaccine or non-vaccines (severe cases = 28; matched controls = 80); 1 case excluded since no matched control was available. ^¶^ Participants who received only the Moderna mRNA-1273 vaccine or non-vaccinees (severe cases = 27; matched controls = 72); 1 case excluded since no matched control was available.

**Table 4:** Protection against any symptomatic COVID-19 disease by complete regimens of vaccine, shown according to time since the second dose *.

		Cases ***		Matched Controls		Protective Effectiveness (PE) % (95% Confidence Interval, CI)			
Vaccines		Vaccinees	Non-Vaccinees	Vaccinees	Non-Vaccinees	Crude PE	*p*-Value	Adjusted ^‡^ PE	*p*-Value
Any vaccine ^†^	≤19 weeks	52	156	199	427	38 (7, 58)	0.02	42 (11, 62) ^€^	0.013
	>19 weeks	90	156	146	466	−91 (−180, −30)	0.001	−35 (−111, 13) ^§^	0.182
Serum Institute of India (ChAdOx1 nCoV-19) **	≤19 weeks	11	148	26	364	8 (−102, 58)	0.827	3 (−129, 59) ^€^	0.940
	>19 weeks	88	156	137	464	−95 (−187, −32)	0.001	−35 (−112, 14) ^§^	0.195
Sinopharm (Vero Cell-Inactivated) ^††^	≤19 weeks	32	153	92	394	24 (−28, 55)	0.303	34 (−16, 62) ^¶^	0.147
	>19 weeks	2	145	2	346	−56 (−1070, 79)	0.664	−36 (−886, 81) ^ƭ^	0.759
Moderna (mRNA-1273) ^‡‡^	≤19 weeks	6	150	42	357	69 (25, 87)	0.010	64 (10, 86) ^ƫ^	0.029

* Received a second dose of vaccine at least 14 days before presentation. ^†^ Any vaccine (Serum Institute of India (ChAdOx1 nCoV-19), Sinopharm (Vero Cell-Inactivated), Moderna (mRNA-1273), and Sinovac (Vero Cell-Inactivated)). ^‡^ Adjusted via a forced variable (severity at presentation) and other covariates significantly associated at *p* < 0.05 in the stepwise model: ^€^ gender, religion, body mass index, and smoking status. ^§^ Gender, religion, household size, and household income. ^¶^ Religion, body mass index, household income, and smoking status. ^ƭ^ Gender, body mass index, and smoking status. ^ƫ^ Gender and smoking status. ** Participants who received only Serum Institute of India-ChAdOx1 nCoV-19 vaccine or non-vaccinees (cases = 270; matched controls = 663); 9 cases excluded since no matched control was available. ^††^ Participants who received only Sinopharm (Vero Cell-Inactivated) vaccinees or non-vaccines (cases = 194; matched controls = 496); 6 cases excluded since no matched control was available. ^‡‡^ Participants who received only Moderna mRNA-1273 or non-vaccines (cases = 165; matched controls = 399); 9 cases excluded since no matched control was available. *** After stratification by time since the second dose, the cases that did not have any matched controls were not included.

## Data Availability

Anonymous participant data and a data dictionary for each variable analyzed in this article, as well as the study protocol, the statistical analysis plan, and the informed consent form will be made available when the study is complete, upon requests directed to the corresponding author (fqadri@icddrb.org). Data can be shared after the approval of a proposal through a secure online platform. The Research Review Committee and Ethical Review Committee at the icddr,b will review the proposal and will then give their approval. Additionally, data sharing will depend on the published data access rules of the icddr,b. Petitioners will need to sign a standard data access agreement issued by the icddr,b.

## Supplementary Materials

The following supporting information can be downloaded at: https://www.mdpi.com/article/10.3390/vaccines10122069/s1, Table S1: Comparability of cases and controls with severe disease at presentation; Table S2: Protection against severe COVID-19 disease at presentation by receipt of complete regimens of vaccines.
