# Supplementary material for: Measuring the Effectiveness of COVID-19 Vaccines Used during a Surge of the Delta Variant of SARS-CoV-2 in Bangladesh: A Test-Negative Design Evaluation
Source: Vaccines (Basel). 2022 Dec 2;10(12):2069. doi: 10.3390/vaccines10122069 (PMC9780914; doi:10.3390/vaccines10122069)
Supplement: Supplementary file 1 [file vaccines-10-02069-s001.zip › TND.Covid.Dhaka.Supplementary_Table_1.pdf]

Supplementary Table S1. Comparability of cases and controls with severe disease at presentation

Name of variables	Parameters	Severe Cases (n=27)	Severe Controls (n=45)	p-value
Month-wise enrolled participant	September	9(33.3%)	8(17.8%)	0.051
	October	11(40.7%)	28(62.2%)	
	November	7(25.9%)	5(11.1%)	
	December	0(0%)	4(8.9%)	
Age in years (mean ± SD)		54.9 ± 18.8	57.3 ± 14.2	0.304
Age groups	18-30 years	2(7.4%)	2(4.4%)	0.491
	31-60 years	15(55.6%)	20(44.4%)	
	61+ years	10(37%)	23(51.1%)	
Sex	Female	18(66.7%)	11(24.4%)	0.001
	Male	9(33.3%)	34(75.6%)	
Religion	Non-Muslim	0(0%)	1(2.2%)	1.000
	Muslim	27(100%)	44(97.8%)	
Body mass index (BMI)	KG/M^2	25.2 ± 4.8	21.7 ± 3.3	0.002
HH members*	Count	4.9 ± 2.1	5.7 ± 2.4	0.126
HH income	BD Taka	27592.6 ± 16380.7	26000 ± 18544.8	0.705
Smokers†	No	25(92.6%)	25(55.6%)	0.002
	Yes	2(7.4%)	20(44.4%)	
Heart disease‡	No	25(92.6%)	38(84.4%)	0.520
	Yes	2(7.4%)	7(15.6%)	
Hypertension‡	No	20(74.1%)	30(66.7%)	0.692
	Yes	7(25.9%)	15(33.3%)	
Lung disease‡	No	27(100%)	44(97.8%)	1.000
	Yes	0(0%)	1(2.2%)	
Diabetes mellitus‡	No	22(81.5%)	39(86.7%)	0.800
	Yes	5(18.5%)	6(13.3%)	
Stomach disease‡	No	27(100%)	45(100%)	-
Kidney disease‡	No	26(96.3%)	45(100%)	0.795
	Yes	1(3.7%)	0(0%)	
Liver disease‡	No	27(100%)	45(100%)	-

Anaemia‡	No	27(100%)	45(100%)	-
Cancer‡	No	26(96.3%)	43(95.6%)	1.000
	Yes	1(3.7%)	2(4.4%)	
Depression‡	No	26(96.3%)	45(100%)	0.795
	Yes	1(3.7%)	0(0%)	
Osteoarthritis‡	No	27(100%)	45(100%)	-
Modified Charlson Comorbidity Index§	Score	1.9 ± 2.4	1.8 ± 2.1	0.891

*HH means household

†A person who smokes tobacco regularly

‡Ascertained by history

§Modified Charlson Comorbidity Index[13]
