# Supplementary material for: Measuring the Effectiveness of COVID-19 Vaccines Used during a Surge of the Delta Variant of SARS-CoV-2 in Bangladesh: A Test-Negative Design Evaluation
Source: Vaccines (Basel). 2022 Dec 2;10(12):2069. doi: 10.3390/vaccines10122069 (PMC9780914; doi:10.3390/vaccines10122069)
Supplement: Supplementary file 1 [file vaccines-10-02069-s001.zip › TND.Covid.Dhaka.Supplementary_Table_2.pdf]

Supplementary Table S2: Protection against severe COVID-19 disease at presentation by receipt of complete regimens of vaccines *

	Severe cases		Severe controls		Protective effectiveness (PE) % (95% confidence interval, CI)			
	Vaccinees	*Non-vaccinees*	*Vaccinees*	*Non-vaccinees*	*Crude PE*	*p-value*	*Adjusted PE*	*p-value*
**Any vaccine**[‡]	3	24	10	35	56(-76,89)	0.244	80 (-3, 98)[ŧ]	0.079
**Serum Institute of India (ChAdOx1 nCoV- 19)**	2	24	1	35	-192(-3300,75)	0.393	-41 (-4220, 96)[ŧ]	0.827
**Sinopharm (Vero Cell-inactivated)**	1	24	6	35	76(-115,97)	0.203	87 (-8, 99)[†]	0.098
**Moderna (mRNA-1273)**	0	24	3	35	100(-Inf,100)	0.991	100(-Inf,100)[ŧ]	0.996

*Received 2nd dose of vaccine at least 14 days before presentation

[†]Adjusted by: forced variables (age in years, study month of presentation, and study site and other covariates which were significantly associated at $p<0.05$ in the stepwise model

[ŧ]Body mass index any, Serum Institute of India, Moderna

[‡]Any vaccine (Serum Institute of India (ChAdOx1 nCoV-19), Sinopharm (Vero Cell- inactivated), Moderna (mRNA-1273))
